# Clinical and laboratory remission with rituximab in anti-MuSK-positive myasthenia gravis

**DOI:** 10.1007/s11845-024-03763-w

**Published:** 2024-08-01

**Authors:** Berin Inan, Irem Gul Orhan, Can Ebru Bekircan-Kurt, Sevim Erdem-Ozdamar, Ersin Tan

**Affiliations:** Department of Neurology, Hacettepe University Faculty of Medicine, Ankara, Turkey

**Keywords:** Monitoring antibody level, Muscle-specific tyrosine kinase, Myasthenia gravis, Rituximab

## Abstract

**Background:**

Increasing data are available on the use and efficacy of rituximab (RTX) in patients with anti-muscle-specific tyrosine kinase (MuSK)-positive myasthenia gravis (MG), especially those steroid-dependent or unresponsive to traditional immunotherapies.

**Aims:**

We aimed to evaluate the clinical characteristics and treatment responses of adult patients with generalized anti-MuSK-positive MG treated with RTX.

**Methods:**

We retrospectively recruited 16 patients who were on RTX, between January 2010 and September 2023. RTX was given 1000 mg/day intravenously twice, two weeks apart. Maintenance treatment was administered at intervals of 3—6 months based on clinical evaluation. The outcome was assessed by Myasthenia Gravis Foundation of America (MGFA) and Myasthenia Gravis Status and Treatment Intensity (MGSTI) scores. Additionally, anti-MuSK antibody levels were retested after treatment in all patients except one.

**Results:**

Twelve patients were female. The mean age at disease onset was 35.3 ± 17.3 years. The median duration between disease onset and RTX administration was 2.4 years (min-max: 0.5-36.5 years). The worst MGFA class before RTX was between IIIb-V. After RTX treatment, 81.3% of patients achieved MGFA minimal manifestations or better and MGSTI level 1 or better. Anti-MuSK antibodies became negative in 12 patients, while they remained positive in three. The changes in antibody levels seemed associated with clinical outcomes.

**Conclusions:**

RTX is an effective treatment in anti-MuSK-positive MG. Furthermore, our results support the inhibition of antibody production by RTX and we recommend monitoring anti-MuSK antibody titers to follow disease progression and treatment response.

## Introduction

Myasthenia gravis (MG) is an antibody-mediated autoimmune disease of neuromuscular junction characterized by fluctuating skeletal muscle weakness and fatigability [1]. Antibodies against acetylcholine receptor are present in 80–90% of MG patients [2], whereas 5–8% of patients have antibodies against muscle-specific tyrosine kinase (MuSK) [3].

The treatment of MG consists of acetylcholinesterase inhibitors, conventional immunosuppressive therapies, fast-acting immunomodulating therapies such as intravenous immunoglobulin (IVIg) and plasmapheresis (PE), and thymectomy [4]. In most cases, MG can be managed effectively. Nevertheless, 10–30% of patients, including patients with anti-MuSK antibodies, are treatment-refractory [5, 6].

Anti-MuSK-positive MG is associated with predominantly bulbar weakness [7, 8]. Clinical onset is usually acute, with rapid progression of symptoms within a few weeks. Myasthenic crisis frequency is higher in anti-MuSK-positive MG than in other MG subtypes [3]. Patients with anti-MuSK-positive MG tend to be unresponsive to acetylcholinesterase inhibitors and more likely to be steroid-dependent despite traditional immunosuppressive agents like azathioprine or mycophenolate mofetil [3, 4]. Recently, rituximab (RTX), a chimeric monoclonal antibody against CD20 transmembrane protein present on the surface of B lymphocytes [9], has emerged as a promising alternative immunosuppressive therapy for these patients based on accumulating data and clinical experience [6, 10–16]. Therefore, in this study, we aimed to evaluate clinical characteristics, treatment responses, and changes in anti-MuSK antibody titers of adult patients with anti-MuSK-positive MG treated with RTX.

## Materials and Methods

We retrospectively recruited adult patients with generalized anti-MuSK-positive MG treated with RTX between January 2010 and September 2023.

Patients were diagnosed based on clinical history, neurological examination, antibody status, and electrodiagnostic testing. The indications for RTX treatment were poor clinical response to other immunosuppressive and immunomodulatory therapies, frequent and/or severe myasthenic exacerbations, and severe side effects from other immunotherapies. RTX was given 1000 mg twice with a 2-week interval as an induction regimen. Maintenance RTX infusions (1000 mg) were administered at intervals of 3–6 months based on the clinical evaluation of each patient.

We evaluated pre and post-RTX symptomatic treatment and immunotherapies, clinical and laboratory findings including demographics, age at disease onset, number of myasthenic exacerbations, disease duration, age at RTX onset, time to relapse, follow-up duration, and anti-MuSK antibody titers. The disease severity and treatment responses were assessed by the Myasthenia Gravis Foundation of America (MGFA) clinical classification [17], MGFA Post-intervention Status (MGFA-PIS) [17], and Myasthenia Gravis Status and Treatment Intensity (MGSTI) scores [12]. Exacerbation was defined as a clinical deterioration requiring rescue treatment or any change in the ongoing treatment regimen.

Baseline anti-MuSK antibody levels were tested by either of the following commercial kits: radioimmunoassay (RIA) (DLD Diagnostika GmbH, Hamburg, Germany) with a cut-off value of 0.05 nmol/L or enzyme-linked immunosorbent assay (ELISA) (IBL International GmbH, Hamburg, Germany) with a cut-off value of 0.4 U/mL. Additionally, we re-tested anti-MuSK antibodies during follow-up in all patients except one, using an ELISA kit (IBL International GmBH, Hamburg, Germany) to assess the effect of RTX treatment.

The institutional and local ethics committees approved the study and waived the requirement for informed consent from patients (2021/05–05—KA21021).

Descriptive statistics were presented as mean ± standard deviation or median (minimum–maximum). Percentages were used for categorical variables. Wilcoxon-signed rank test or Friedman test for paired analysis were used for non-parametric data. Statistical analysis was performed using IBM SPSS software (SPSS, Inc, Chicago, IL. USA, version 23), and significance was defined as a *p*-value of < 0.05.

## Results

Demographic, clinical, and laboratory characteristics of the patients are presented in detail in Table 1.

**Table 1:** Demographic, clinical, and laboratory characteristics of the patients

Case #, sex	Age at disease onset (y)	Age at RTX onset (y)	Before RTX					After RTX						anti-MuSK ab levels	
			# of myasthenic exacerbations	Previous treatments	Treatments at RTX onset	Worst MGFA class	MGSTI level (last three months)	# of myasthenic exacerbations	# of RTX cycles	Follow-up duration (mo)	MGFA-PIS (last visit)	MGSTI level (last visit)	Treatments at last visit	Baseline	Post-RTX
1, F	17	20	4	PD, MPZ, IVIg	PD, MPZ, IVIg	IVb	6	1	4	56	CSR	0	-	> 12 U/mL	Negative
2, F	20	24	4	PD, MPZ	PD, MPZ	IIIb	2	None	3	54	CSR	0	-	1.76 nmol/L^a^	Negative
3, F	22	29	15	PD, MPZ, MMF, IVIg	PD, MPZ, MMF	IVb	3	None	3	49	CSR	0	-	1.46 nmol/L^a^	Negative
4, M	26	62	37	PD, DFZ, MPZ, IVIg	PD, MPZ	IVb	4	None	6	51	Improved	1	-	> 12 U/mL	Negative
5, F	24	27	4	PD, MPZ, AZA, IVIg	PD, MPZ, AZA, IVIg	IVb	6	None	3	57	CSR	0	-	2.54 nmol/L^a^	Negative
6, F	16	25	6	PD, MPZ, MMF, IVIg	MPZ	IVb	2	None	2	58	CSR	0	-	12 U/mL	Negative
7, M	57	58	2	PD, MPZ, IVIg	MPZ	IVb	6	None	2	38	CSR	0	-	12 U/mL	Negative
8^b^, F	26	27	5	PD, MPZ, IVIg, PE	MPZ, PE	V	6	10	9	78	Unchanged	5	Intermittent PE	1.79 nmol/L^a^	NA
9, F	65	66	2	PD, Pred, IVIg	PD, Pred, IVIg	IVb	6	1 + 1	4 + 1	50	MM1	2	MPZ	5.7 U/mL	11.5 U/mL
10, F	63	65	2	PD, MPZ, AZA, IVIg	PD, MPZ, AZA, IVIg	IIIb	6	None	8	53	CSR	0	-	11.4 U/mL	1.9 U/mL
11, F	41	43	3	PD, Pred, AZA, IVIg	PD, Pred, AZA, IVIg	IVb	6	None	6	54	CSR	0	-	1.49 nmol/L^a^	Negative
12, F	45	46	5	PD, Pred, AZA, IVIg	PD, Pred, AZA	V	6	1	5	64	CSR	0	-	> 12 U/mL	Negative
13, M	16	19	5	PD, Pred, CsA, IVIg	PD, CsA, IVIg	V	5	1	5 + 2	54	Improved	4	PD	2.41 nmol/L^a^	3.4 U/mL
14, F	52	63	6	PD, Pred, AZA, IVIg	Pred, AZA, IVIg	IVb	5	None	6	48	CSR	0	-	1.85 nmol/L^a^	Negative
15, M	46	47	1	PD, MPZ	PD, MPZ	IIIb	4	1	3	54	CSR	0	-	9.8 U/mL	Negative
16, F	29	31	2	PD, DFZ, AZA	DFZ, AZA	IVb	4	None	3	44	CSR	0	-	7.3 U/mL	Negative

^#^ = number, F = female, M = male, MuSK = muscle-specific tyrosine kinase, ab = antibody, RTX = rituximab, IVIg = intravenous immunoglobulin, PE = plasma exchange, PD = pyridostigmine, MPZ = methylprednisolone, DFZ = deflazacort, Pred = prednisolone, MMF = mycophenolate mofetil, AZA = azathioprine, CsA = cyclosporine A, MGSTI = Myasthenia Gravis Status and Treatment Intensity, MGFA = Myasthenia Gravis Foundation of America, PIS = post-intervention status, CSR = complete stable remission, MM = minimal manifestations, NA = not available

^a^Tested with radioimmunoassay of DLD Diagnostika GmbH, Hamburg, Germany (titer > 0.05 nmol/L is positive). The remaining anti-MuSK antibodies were tested with an enzyme-linked immunosorbent assay of IBL International GmbH, Hamburg, Germany (titer > 0.4 U/mL is positive)

^b^Case 8 underwent autologous hematopoietic stem cell transplantation for treatment-refractory MG and published as a case report (Inan B et al. (2022) Autologous stem cell transplantation in a patient with refractory anti-MuSK-Positive myasthenia gravis and familial mediterranean fever. Neurological Sciences and Neurophysiology 39:115–118.)

Sixteen MG patients were included in the study. Twelve (75%) of them were female. The mean age at disease onset was 35.3 ± 17.3 years. The median duration between disease onset and RTX administration was 2.4 years (min–max: 0.5–36.5 years).

The mean number of RTX cycles (including induction and maintenance) was 4.7 ± 2.2. RTX was well-tolerated, no side effects were observed.

Two patients (Case 4 and 13) had persistent nasal speech and tongue atrophy prior to RTX treatment. RTX was discontinued in Case 4, whose anti-MuSK antibody level became negative. Case 13 remained relapse-free during five cycles of RTX. Eighteen months after discontinuing RTX treatment he exhibited a relapse characterized by bulbar symptoms that did not require ventilatory support, leading to the resumption of RTX treatment.

RTX made no substantial changes in Case 8, who experiences frequent myasthenic exacerbations. She was a challenging patient with a history of familial Mediterranean fever (FMF), steroid-induced myopathy and severe fasciculations due to pyridostigmine. Moreover, she could not use other immunotherapies such as azathioprine and intermittent IVIg because of severe side effects such as leukopenia and thrombosis. Finally, she underwent autologous hematopoietic stem cell transplantation for MG with a modest response. Currently, she is still on RTX and intermittent plasmapheresis [18].

Lastly, Case 9 maintained clinical stability after completing four cycles of RTX, which allowed for the discontinuation of all immunosuppressive treatments. Nevertheless, three years after the cessation of treatment, she experienced a second myasthenic relapse, RTX and MPZ treatments were resumed. Meanwhile, her anti-MuSK antibody titer were higher than the initial titer.

The mean length of follow-up after RTX treatment was 53.9 ± 8.7 months (4.8 ± 0.8 years). Corticosteroids were tapered off in 4 of 15 patients and reduced to a median daily dose of 30 mg (min–max: 5–60 mg) of prednisone or equivalent in 11 patients at 6 months. For all 15 patients, the median daily dose of prednisone or equivalent decreased from 40 mg (min–max: 7.5–80.0 mg) at baseline to 15 mg (min–max: 0–60 mg) at 6 months (*p* = 0.003). At the last visit, 93% of patients withdrew corticosteroids, and only one patient (Case 9) was taking a daily dose of 10 mg prednisolone (*p* < 0.001). The median time to withdraw corticosteroids after RTX treatment was 11 months (min–max: 4–35, mean 14.1 ± 10.1).

Thirteen (81.3%) patients achieved MGFA-PIS minimal manifestations (MM) or better and reached MGSTI level 1 or better at the last visit. Anti-MuSK antibodies became negative in 12 of 15 patients (80%) who had anti-MuSK antibody levels retested after RTX treatment.

## Discussion

This is a retrospective study of a cohort of 16 patients with anti-MuSK-positive MG treated with RTX in a single tertiary healthcare center.

Our cohort was similar to other anti-MuSK-positive MG cohorts in terms of sex [3, 7, 11, 15, 19, 20] and age at RTX initiation [11, 15]. Nevertheless, our cohort also included two patients over 65 years old at RTX initiation. The optimal management of MG in older individuals (≥ 65 y) is unknown [21] and safety and efficacy concerns may arise due to comorbidities, polypharmacy, and frailty. However, RTX was efficient and well-tolerated in these patients without significant adverse events consistent with previous studies [13, 21].

There is currently no consensus for RTX regimen in MG [9, 22, 23]. Protocols for RTX induction and maintenance therapy vary among different medical centers [6, 7, 13, 14, 20–22]. Some authors have suggested that more aggressive treatment protocols yield higher rates of clinical remission [24], a more durable response, and lower relapse rates [7, 14], while others argue that low doses of RTX are as effective as standard induction doses [6, 20, 22, 25–28]. In our study, we administered two infusions of 1000 mg RTX two weeks apart for induction, following one of the routine induction protocols.

The ratio of patients reaching MGFA-PIS MM or better after RTX treatment varies between 50 and 100% [6, 7, 11–15, 20, 22, 29, 30], and the percentage of patients who are able to withdraw oral immunosuppressants, including corticosteroids, is between 22–97% [6, 7, 12, 29, 31] in previous studies. The majority (81.3%) of the patients achieved MGFA-PIS MM or better in our cohort. In contrast, two patients (Case 4 and 13) had less satisfactory treatment responses, and a unique patient (Case 8) with a history of FMF and severe side effects to multiple treatments had limited response to RTX. She required autologous stem cell transplantation, which showed modest improvement in her myasthenic symptoms [18]. Furthermore, 93% of patients discontinued corticosteroids, and all patients taking other oral immunosuppressants withdrew their medications. The sample size was small, so we could not statistically analyze the factors predicting clinical response. However, based on our clinical observation, we may suggest that the higher the frequency of myasthenic exacerbations and the longer the disease duration, the worse the RTX response. Thus, RTX may be better in controlling relapses and achieving remission when started earlier without the disease severely worsening.

Anti-MuSK antibodies were retested during follow-up in 15 of 16 patients. Eleven of twelve patients who became antibody-negative reached MGSTI Level 0, and MGFA-PIS CSR. The remaining patient (Case 4) also improved and discontinued all immunotherapies. However, he still has sequelae bulbar findings such as tongue atrophy because of the long disease duration. Three patients, who remained antibody-positive, achieved MGSTI level 0, 2 and 4 (MGFA-PIS CSR, MM1, and improved respectively). Thus, changes in antibody levels seemed associated with clinical outcomes. Similarly, striking reduction in anti-MuSK antibody titers and anti-MuSK antibodies turning negative were previously reported in patients clinically responding to RTX [7, 20, 30, 31]. In addition, the changes in levels of anti-MuSK antibodies were clinically correlated in all studies [7, 20, 30, 31]. RTX is efficient and helps taper off corticosteroids and other immunosuppressants in anti-MuSK MG. RTX depletes all immature and mature B cells, memory B cells [26] but has a limited effect on plasmablasts and plasma cells, the B cell subtypes producing antibodies [26, 32]. This means RTX has a selective impact on short-lived plasma cells [7], and as the long-lived plasma cell pool increase during the disease course, the therapeutic effect of RTX diminishes [15, 26]. Thus, as recently proposed in a randomized clinical trial [27], we suggest that RTX might be considered an early therapeutic option, even as a first-line treatment to reduce the risk of disease worsening in anti-MuSK positive generalized MG. We also highly recommend monitoring anti-MuSK antibody titers to follow disease progression and treatment response.

There are some limitations of our study, mostly stemming from its retrospective design. First, baseline and follow-up anti-MuSK antibodies were tested with different assays. Thus, we could not compare the positive results in baseline and follow-up samples of some patients due to the difference in the assays. Second, the sampling intervals were not fixed. The samples were taken at variable time points after variable cycles of RTX due to the retrospective design of our study. Lastly, the sample size was small. Prospective studies in larger cohorts, with predefined treatment regimens and certain sampling intervals, are warranted to elucidate the effect of RTX treatment on anti-MuSK antibody levels and the association between disease course and antibody levels.

## Data Availability

Data are available from the corresponding author upon reasonable request.
